# Androgen deprivation therapy plus abiraterone or docetaxel as neoadjuvant therapy for very-high-risk prostate cancer: a pooled analysis of two phase II trials

**DOI:** 10.3389/fphar.2023.1217303

**Published:** 2023-06-26

**Authors:** Junlong Zhuang, Yuwen Wang, Shun Zhang, Yao Fu, Haifeng Huang, Xiaoyu Lyu, Shiwei Zhang, Giancarlo Marra, Linfeng Xu, Xuefeng Qiu, Hongqian Guo

**Affiliations:** ^1^ Department of Urology, Affiliated Drum Tower Hospital, Medical School of Nanjing University, Nanjing, China; ^2^ Institute of Urology, Nanjing University, Nanjing, China; ^3^ Medical School of Southeast University Nanjing Drum Tower Hospital, Nanjing, China; ^4^ Department of Pathology, Affiliated Drum Tower Hospital, Medical School of Nanjing University, Nanjing, China; ^5^ Department of Surgical Sciences, Division of Urology, Molinette Hospital, Città della Salute e della Scienza and University of Turin, Turin, Italy

**Keywords:** prostate cancer, neoadjuvant therapy, abiraterone, docetaxel, radical prostatectomy

## Abstract

**Objective:** The study aimed to compare the efficacy and safety of androgen deprivation therapy (ADT) with abiraterone or docetaxel versus ADT alone as neoadjuvant therapy in patients with very-high-risk localized prostate cancer.

**Methods:** This was a pooled analysis of two single-center, randomized, controlled, phase II clinical trials (*ClinicalTrials.gov*: NCT04356430 and NCT04869371) conducted from December 2018 to March 2021. Eligible participants were randomly assigned to the intervention (ADT plus abiraterone or docetaxel) and control (ADT alone) groups at a 2:1 ratio. Efficacy was evaluated by pathological complete response (pCR), minimal residual disease (MRD), and 3-year biochemical progression-free survival (bPFS). Safety was also analyzed.

**Results:** The study included 42 participants in the ADT group, 47 in the ADT plus docetaxel group, and 48 in the ADT plus abiraterone group. A total of 132 (96.4%) participants had very-high-risk prostate cancer, and 108 (78.8%) had locally advanced disease. The ADT plus docetaxel group (28%) and ADT plus abiraterone group (31%) had higher rates of pCR or MRD (*p* = 0.001 and *p* < 0.001) compared with the ADT group (2%). The 3-year bPFS was 41.9% (95% CI: 26.6–57.2), 51.1% (95% CI: 36.8–65.4), and 61.2% (95% CI: 45.5–76.9), respectively. Significant difference was found among groups in terms of bPFS (*p* = 0.037).

**Conclusion:** Compared with ADT alone, neoadjuvant therapy with ADT plus docetaxel or abiraterone could achieve better pathological outcomes (pCR or MRD) for very-high-risk localized prostate cancer. The ADT plus abiraterone group showed longer bPFS than ADT alone. The combination regimens were tolerable.

## 1 Introduction

Newly diagnosed prostate cancer (PCa) varies considerably in its clinical aggression and, therefore, in the preferred initial management strategy (Reese et al., 2012). Men with high-risk PCa (HRPCa) characterized by aggressive pathological grade, advanced T stage, or a high level of prostate-specific antigen (PSA) were at a higher risk of treatment failure, oncological progression, and local or systematic recurrence (Chang et al., 2014; Wilkins et al., 2020). However, HRPCa is still a highly heterogeneous group with a wide range of prognoses. Biochemical recurrence (BCR) rates among high-risk PCa can vary by over 50% at 10 years (Sundi et al., 2019). Ten-year metastasis-free survival (MFS) can range from 70% to 95% depending on pathoclinical characteristics among high-risk men who undergo radical prostatectomy (RP) (Loeb et al., 2010). Patients with very-high-risk prostate cancer (VHRPCa) have worse postoperative pathological outcomes including positive margins and positive lymph nodes (Mano et al., 2016; Sundi et al., 2019). Moreover, patients with VHRPCa are at higher risks of metastasis (nearly three times higher than HRPCa) and cancer-specific mortality (nearly seven times higher than HRPCa) (Sundi et al., 2019). A different management might be in demand for subgroups with different malignant potential.

Since radical prostatectomy monotherapy proved insufficient, the combination of neoadjuvant systemic therapy and radical prostatectomy (RP) has been applied to the multi-modality therapy of HRPCa (Sanda et al., 2018; Boyle et al., 2019). Meta-analyses demonstrated that the neoadjuvant ADT could lower the risk of extracapsular extension, positive surgical margin, and lymph node metastasis after RP (Katayama et al., 2022; Kishan et al., 2022). Nevertheless, compared with RP alone, neoadjuvant ADT therapy failed to show statistically significant improvements in biochemical progression-free survival (bPFS) and overall survival (OS) (Schulman et al., 2000; McKay et al., 2018).

The tremendous advances in metastatic prostate cancer treatment with new-generation hormone therapies and docetaxel-based chemotherapy also provide new opportunities for neoadjuvant treatment of HRPCa (Tannock et al., 2004; Virgo et al., 2021; NCCN Clinical Practice Guidelines in Oncology NCCN Guidelines, 2022). It is hypothesized that these benefits might apply better to earlier stages of PCa as a more homogeneous cancer cell population is expected in the localized setting (Devos et al., 2021). Recent phase II trials explored the efficacy of ADT plus abiraterone or docetaxel as neoadjuvant therapy in HRPCa, proving that such treatment is safe and viable with objective clinical application prospects (Taplin et al., 2014; Thalgott et al., 2014; Efstathiou et al., 2019). However, these trials rarely enrolled patients with VHRPCa.

Although available data suggest promising efficacy of the ADT-based combination of neoadjuvant regimens, evidence remains insufficient, especially for VHRPCa. Therefore, a pooled analysis of two phase II clinical trials was carried out in very-high-risk PCa (VHRPCa) with the purpose of comparing the efficacy and safety of neoadjuvant ADT plus abiraterone or docetaxel to ADT alone.

## 2 Materials and methods

### 2.1 Study design and patients

This study was a pooled analysis of two single-center, randomized controlled phase II clinical trials (*ClinicalTrials.gov*: NCT04356430 and NCT04869371) conducted from December 2018 to March 2021, complying with the Good Clinical Practice (GCP), Declaration of Helsinki, relevant regulations, and ethics committee approval. All patients provided written informed consent.

Main inclusion criteria are as follows: 1) male subjects of 18–75 years of age; 2) diagnosis of PCa by biopsy and eligible for RP; 3) high-risk or very-high-risk PCa that met one of the following criteria: a) multi-parameter magnetic resonance imaging (mpMRI) and ^68^Ga prostate-specific membrane antigen (PSMA)-ligand positron emission tomography (PET)/computed tomography (CT) (^68^Ga PSMA-PET/CT) scan indicating primary tumor staging ≥T3; b) biopsy Gleason score ≥ 8; and c) serum PSA > 20 ng/mL.

The risk is finally stratified according to NCCN Guidelines for Prostate Cancer (Version 4.2022). HRPCa was defined as having no very-high-risk features and having exactly one high-risk feature: clinical T3a, or grade group 4 or 5, or PSA > 20 ng/mL. VHRPCa was defined as meeting at least one of the following criteria: clinical T3b–T4, or primary Gleason pattern 5, or >4 cores with grade group 4 or 5, or 2–3 high-risk features.

Main exclusion criteria are as follows: 1) neuroendocrine, small-cell, or sarcomatoid features of prostate histopathology; 2) clinical or radiological evidence of regional or extra-regional lymph node metastasis or metastasis of bone or viscera (any N1 or M1); 3) previously treated with ADT, radiotherapy, or chemotherapy for PCa; and 4) patients with severe or uncontrollable chronic or infectious disease, or other malignant tumors within 5 years.

### 2.2 Neoadjuvant intervention

In the two trials, eligible participants were randomly assigned to the experimental (ADT plus docetaxel or ADT plus abiraterone) and control (ADT alone) groups at a ratio of 2:1. All participants received hypodermic injection of luteinizing hormone-releasing hormone analog (LHRHa) every 12 weeks. Furthermore, participants in the ADT plus abiraterone group also took 1,000 mg of abiraterone acetate and 5 mg of prednisone acetate daily. Participants in the ADT plus docetaxel group additionally administered docetaxel intravenously at 75 mg/m^2^ body surface area every 3 weeks for six cycles in addition to 5 mg of prednisone acetate twice a day.

All participants received 24 weeks of neoadjuvant therapy followed by robot-assisted radical prostatectomy (RARP) and extended pelvic lymph node dissection (ePLND) within 2 weeks after the end of the therapy.

### 2.3 Follow-up

Within 7 days after the completion of neoadjuvant therapy, the following scans were performed: MRI 3.0-T scan of the prostate, PSMA-PET/CT scan or emission CT (ECT) bone scan, and CT scan for the chest and whole abdomen. PSA levels were examined every month after surgery. The follow-up was terminated at biochemical progression or the patients’ withdrawal of informed consent, whichever happened first.

### 2.4 Outcomes and definitions

Primary outcomes included are as follows: 1) pathological complete response (pCR) rate and 2) pCR or minimal residual disease (MRD) rate. pCR was defined as the absence of morphologically recognizable carcinoma in the prostatectomy specimen. MRD was defined as the maximum diameter of residual tumor in the large pathological section of prostatectomy ≤5 mm.

Secondary outcomes mainly included the following: 1) 3-year biochemical progression-free survival (bPFS); biochemical progression is defined as two consecutive rising PSA values that are above 0.2 ng/mL at least 1 month apart, or initiation of adjuvant therapy after surgery including radiotherapy, ADT, or anti-androgen therapy. The time for bPFS was measured from randomization to biochemical progression or death from any cause. 2) The serum complete response rate is defined as the proportion of participants with PSA ≤ 0.1 ng/mL after 6-month neoadjuvant therapy.

Other outcomes included the down-staging ratio of specimen pathology, rate of positive margins, rate of extracapsular extension, N staging, and tumor volume after neoadjuvant therapy assessed by images.

All adverse events (AEs) were carefully recorded according to MedDRA and graded according to NCI CTCAE 5.0. Serious AEs (SAEs).

### 2.5 Statistical analysis

In each trial, it was determined that 75 participants would provide approximately 80% power for the assumed pCR or MRD rate of 30% in the combination treatment group and 5% in the control group when a 20% of dropout rate was taken into account. Two-sided 95% confidence intervals were calculated for the primary and secondary endpoints. The pooled analysis combined the control groups of the two trials as the ADT group. Patients who completed full intervention cycles, underwent RP, and completed the follow-up were included for efficacy analysis. All patients’ safety outcomes were analyzed.

SPSS 22.0 (IBM, Armonk, NY, United States) was used for statistical analysis. Mean ± standard deviation and median (interquartile range) were used to describe continuous variables. ANOVA or the Kruskal–Wallis test was used for multi-group comparison. The categorical variables were described as frequency (percentage). Fisher’s exact test and Pearson’s chi-squared test were used for multi-group comparison. Bonferroni correction was applied for pairwise comparison after multi-group comparison. Multivariable logistic regression analysis explored the independent factors influencing the efficacy outcome (pCR/MRD). Tests were performed to confirm the proportional hazard assumption for bPFS. Kaplan–Meier analysis was used to analyze bPFS, and the log-rank test was conducted to compare groups. Multivariable Cox regression analysis was used to probe into the independent factors influencing bPFS. The significant level was set at a *p*-value <0.05.

## 3 Results

### 3.1 Characteristics of the participants

**FIGURE 1:**
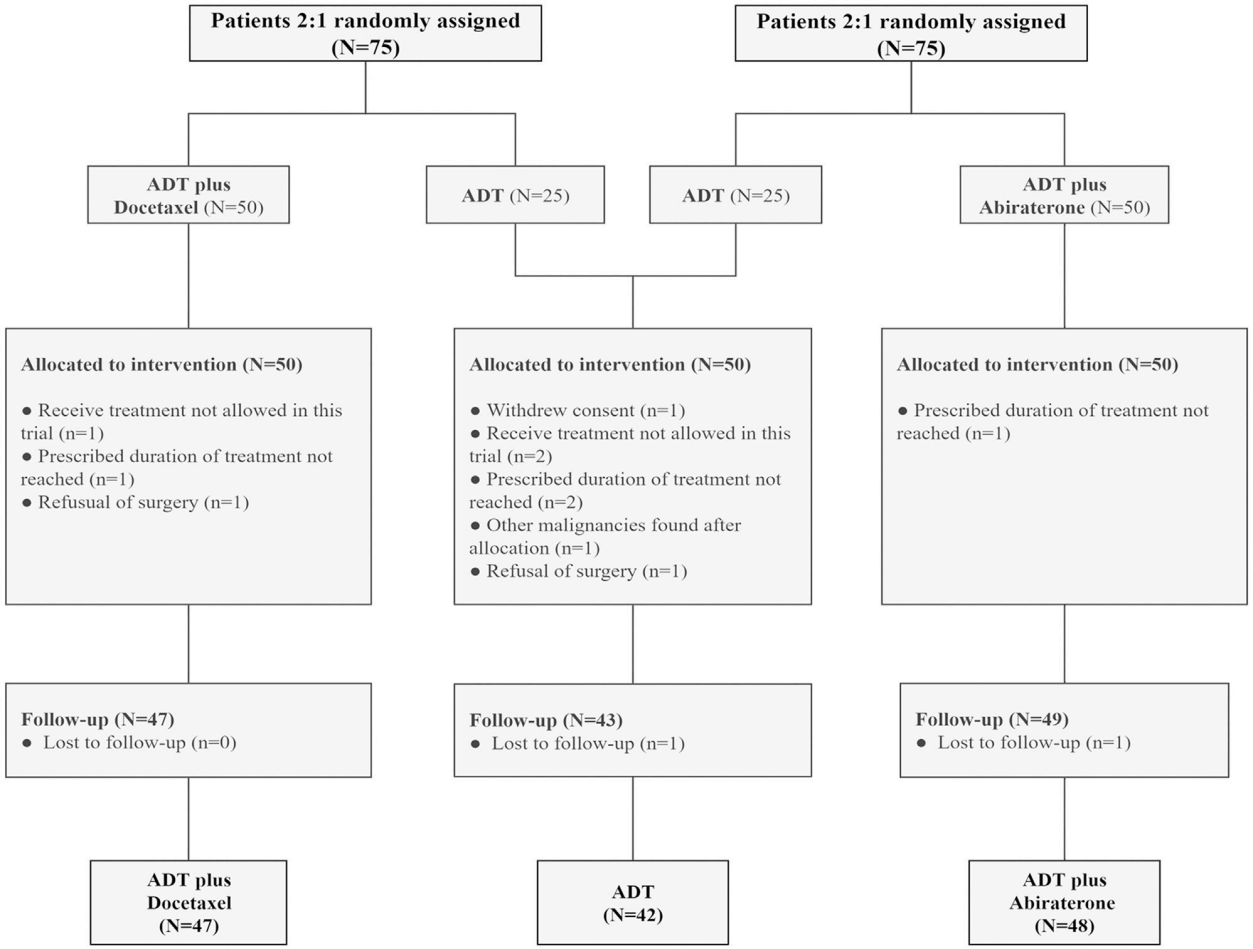
Consort diagram: patients with high-risk and very-high-risk PCa in each trial were randomly assigned at a ratio of 2:1 to the intervention (ADT plus abiraterone or docetaxel) and control (ADT alone) groups. Thirteen patients were excluded. ADT, androgen deprivation treatment.

Each trial enrolled 75 participants, including 50 in the combined therapy group and 25 in the control group. The pooled analysis included 42 participants in the ADT group, 47 in the ADT plus docetaxel group, and 48 in the ADT plus abiraterone group. Thirteen patients were excluded (Figure 1). Baseline characteristics were well-balanced among three groups, such as age, initial PSA, and Gleason score (Table 1). A total of 132 (96.4%) participants had very-high-risk prostate cancer, and 108 (78.8%) had locally advanced disease (clinical T3 or T4).

**TABLE 1:** Baseline characteristics. Age was evaluated by one-way ANOVA. Initial PSA, initial volume evaluated by the Kruskal–Wallis test. Biopsy Gleason score was evaluated by Fisher’s exact test. Initial T stage and risk stratification were evaluated by Pearson’s chi-squared test.

Characteristic	ADT (N = 42)	ADT plus docetaxel (N = 47)	ADT plus abiraterone (N = 48)	*p*-value
Age, years				0.150
Mean ± SD	69.2 ± 3.7	67.0 ± 6.8	68.5 ± 5.6	
Median (IQR)	70 (67–72)	69 (62–73)	70 (64–73)	
Initial PSA, ng/mL				0.803
Mean ± SD	54.8 ± 44.2	71.7 ± 93.6	54.6 ± 71.7	
Median (IQR)	44.1 (23.9–62.5)	34.1 (17.4–80.0)	38.1 (17.6–60.9)	
Biopsy Gleason score, n (%)				0.571
3 + 3 = 6	2 (5)	1 (2)	1 (2)	
3 + 4 = 7	3 (7)	0	3 (6)	
4 + 3 = 7	7 (17)	8 (17)	13 (27)	
4 + 4 = 8	16 (38)	24 (51)	20 (42)	
4 + 5 = 9	9 (21)	8 (17)	9 (19)	
5 + 4 = 9	4 (10)	3 (6)	1 (2)	
5 + 5 = 10	1 (2)	3 (6)	1 (2)	
Initial T stage, n (%)				0.077
T2	13 (31)	6 (13)	10 (21)	
T3a	10 (24)	18 (38)	7 (15)	
T3b	15 (36)	17 (36)	21 (44)	
T4	4 (10)	6 (13)	10 (21)	
Initial volume, mL				0.993
Mean ± SD	37.2 ± 13.4	38.5 ± 18.4	39.1 ± 17.1	
Median (IQR)	33.7 (29.0–42.0)	35.7 (26.2–47.5)	34.4 (25.3–51.9)	
Risk stratification, n (%)				0.442
High	3 (7)	1 (2)	1 (2)	
Very high	39 (93)	46 (98)	47 (98)	

ADT, androgen deprivation therapy; SD, standard deviation; IQR, interquartile range; PSA, prostate-specific antigen.

### 3.2 Pathological outcomes

In terms of the pCR rate, there were significant differences among three groups (*p* = 0.013). Compared with the ADT group (0%), the ADT plus abiraterone (19%) and ADT plus docetaxel (17%) groups demonstrated a significant advantage (*p* = 0.003 and *p* = 0.006, respectively). In terms of pCR or MRD rates, significant differences were also observed among three groups (*p* = 0.002); the ADT plus abiraterone (31%) and ADT plus docetaxel (28%) groups also had obvious advantages (*p* < 0.001 and *p* = 0.001, respectively) compared with the ADT group (2%). Although differences were not significant, the combined therapy group lowered the risk of positive margins, pathological N1, and extraprostatic extension while increasing the rate of pathological downgrade (Table 2).

**TABLE 2:** Post-treatment pre-RP and pathological outcomes. All except the post-treatment volume was evaluated by Pearson’s chi-squared test. The post-treatment volume was evaluated by the Kruskal–Wallis test.

Characteristic	ADT (N = 42)	ADT plus docetaxel (N = 47)	ADT plus abiraterone (N = 48)	*p*-value
Post-treatment outcomes				
Tumor volume, mL^a^				0.002**
Mean ± SD	21.3 ± 9.1	20.6 ± 8.2	15.9 ± 5.8	
Median (IQR)	20.7 (14.1–25.1)	18.2 (15.1–23.6)	15.3 (12.1–18.5)	
PSA, ng/mL^b^				<0.001**
≤0.1, n (%)	12 (29)	18 (38)	40 (83)	
>0.1, n (%)	30 (71)	29 (62)	8 (17)	
Pathological outcomes				
pCR, n (%)^c^	0	8 (17)	9 (19)	0.013*
MRD, n (%)	1 (2)	5 (11)	6 (13)	0.203
pCR or MRD, n (%)^d^	1 (2)	13 (28)	15 (31)	0.002**
Pathological downgrade, n (%)	19 (45)	28 (60)	30 (63)	0.219
Positive margin, n (%)	10 (24)	10 (21)	9 (19)	0.842
Pathological N1, n (%)	7 (17)	3 (6)	9 (19)	0.179
Extraprostatic extension, n (%)	26 (62)	22 (47)	22 (46)	0.242
T3a, n (%)	16 (38)	10 (21)	9 (19)	
T3b, n (%)	9 (21)	12 (26)	13 (27)	
T4, n (%)	1 (2)	0	0	

*p < 0.05.

**p < 0.01.

^a^ ADT vs. ADT plus abiraterone group: *p* = 0.002; ADT plus docetaxel vs. ADT plus abiraterone group: *p* = 0.003; and ADT vs. ADT plus docetaxel group: *p* = 0.808.

^b^ ADT vs. ADT plus abiraterone group: *p* < 0.001; ADT plus docetaxel vs. ADT plus abiraterone group: *p* < 0.001; and ADT vs. ADT plus docetaxel group: *p* = 0.333.

^c^ ADT vs. ADT plus abiraterone group: *p* = 0.003; ADT vs. ADT plus docetaxel group: *p* = 0.006; and ADT plus docetaxel vs. ADT plus abiraterone group: *p* = 0.826.

^d^ ADT vs. ADT plus abiraterone group: *p* < 0.001; ADT vs. ADT plus docetaxel group: *p* = 0.001; and ADT plus docetaxel vs. ADT plus abiraterone group: *p* = 0.701.

ADT, androgen deprivation therapy; RP, radical prostatectomy; SD, standard deviation; IQR, interquartile range; pCR, pathological complete response; MRD, minimal residual disease.

The independent predictors for pCR or MRD included ADT plus abiraterone (OR = 16.66, 95% CI: 1.86–149.43, and *p* = 0.012), ADT plus docetaxel (OR = 16.29, 95% CI: 1.88–141.05, and *p* = 0.011) (ADT as a reference), Gleason score (OR = 2.11, 95% CI: 1.31–3.41, and *p* = 0.002), and pre-operative PSA ≤ 0.1 ng/mL (OR = 3.75, 95% CI: 1.04–13.50, *p* = 0.044, and PSA > 0.1 ng/ml as a reference) (Table 3).

**TABLE 3:** Logistic regression model for pCR or MRD.

Factor	pCR or MRD	
	OR (95% CI)	*p*-value
Group		
ADT plus docetaxel vs. ADT	16.29 (1.88–141.05)	0.011*
ADT plus abiraterone vs. ADT	16.66 (1.86–149.43)	0.012*
Gleason^a^	2.11 (1.31–3.41)	0.002**
Post-treatment pre-RP PSA (≤0.1 vs. >0.1)	3.75 (1.04–13.50)	0.044*

*p < 0.05.

**p < 0.01.

^a^ Gleason enters the model as a continuous variable.

pCR, pathological complete response; MRD, minimal residual disease; OR, odds ratio; CI, confidence interval; ADT, androgen deprivation therapy.

### 3.3 Clinical efficacy

**FIGURE 2:**
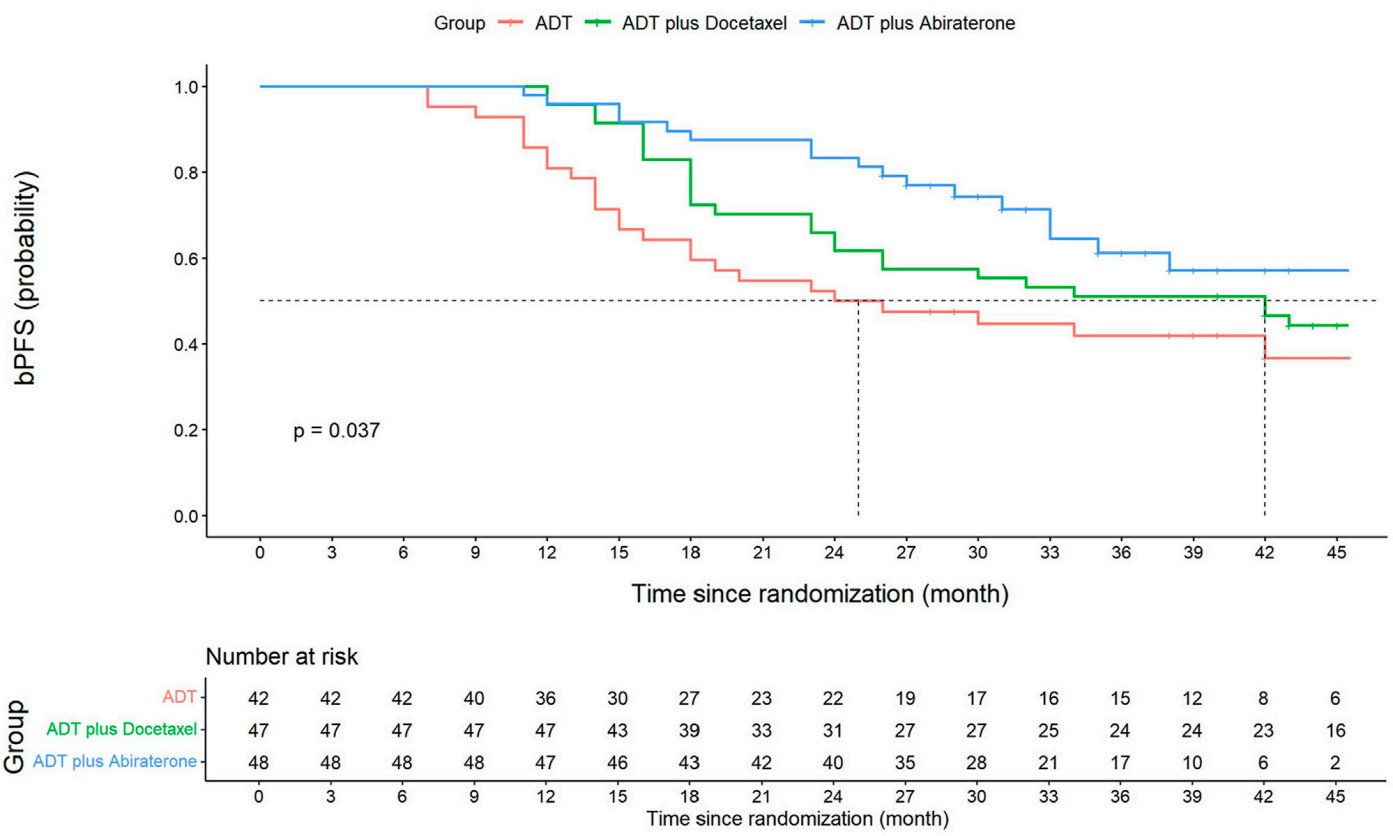
Kaplan–Meier graph of biochemical progression-free survival (bPFS). Three-year bPFS at 41.9% (95% CI: 26.6–57.2) for the ADT group, 51.1% (95% CI: 36.8–65.4) for the ADT plus docetaxel group, and 61.2% (95% CI: 45.5–76.9) for the ADT plus abiraterone group. Significant differences were found among three groups in terms of bPFS (log-rank *p* = 0.037). ADT vs. ADT plus docetaxel, log-rank *p* = 0.199. ADT vs. ADT plus abiraterone, log-rank *p* = 0.013*. ADT plus docetaxel vs. ADT plus abiraterone, log-rank *p* = 0.177. The overall bPFS at 1, 2, and 3 years is 91.2% (95% CI: 86.5–95.9), 65.7% (95% CI: 57.7–73.7), and 52.4% (95% CI: 43.8–61.0), respectively. The 1-year and 2-year bPFS for the ADT group is 81.0% (95% CI: 74.9–87.1) and 50.0% (95% CI: 57.7–42.2), respectively. The 1-year and 2-year bPFS for the ADT plus docetaxel group is 95.7% (95% CI: 92.8–98.6) and 61.7% (95% CI: 54.6–68.8), respectively. The 1-year and 2-year bPFS for ADT plus abiraterone group is 95.8% (95% CI: 93.7–97.9) and 83.3% (95% CI: 77.9–88.7), respectively.

The median follow-up time for the ADT, ADT plus docetaxel, and ADT plus abiraterone groups was 40, 48, and 35 months, respectively, with the overall median follow-up time of 42 months. The 3-year bPFS was 41.9% (95% CI: 26.6–57.2), 51.1% (95% CI: 36.8–65.4), and 61.2% (95% CI: 45.5–76.9) for three groups, respectively. Significant differences were found among groups in terms of bPFS (*p* = 0.037) (Figure 2). According to the pairwise comparison, the ADT plus abiraterone group proved to be more beneficial than the ADT group (*p* = 0.013) but showed no significant difference compared with the ADT plus docetaxel group (*p* = 0.177). The ADT plus docetaxel group showed no significant advantage over the ADT group (*p* = 0.199) in bPFS.

According to multivariate Cox analysis, ADT plus abiraterone (HR = 0.44, 95% CI: 0.23–0.83, and *p* = 0.011; in comparison with the ADT group) was conducive to achieving a better bPFS, whereas ADT plus docetaxel was not a facilitator (HR = 0.64, 95% CI: 0.37–1.11, and *p* = 0.110). On the other hand, positive margins and a more advanced pathological T stage were risk factors for poor bPFS (Supplementary Table S1).

Post-biochemical progression treatments are shown in Supplementary Table S3. Testosterone level recovery and post-RARP urinary continence recovery are displayed in Supplementary Figure S1 and Supplementary Figure S2. Supplementary Table S4 presents pre-treatment and post-treatment 68Ga-PSMA-11 PET/CT-related information, and Supplementary Table S5 shows its correlation with pCR.

### 3.4 Safety

Adverse events occurred at 81% in the ADT plus abiraterone group, 89% in the ADT plus docetaxel group, and 68% in the ADT group. As is shown in Supplementary Table S2, hypokalemia (56%), hot flashes (50%), and hyperglycemia (38%) were the most common AEs for the ADT plus abiraterone group. Granulocytopenia (74%), anemia (50%), and hot flashes (44%) were the most common AEs for the ADT plus docetaxel group. Hot flashes (46%) were the most common AEs in the ADT group.

AEs with grade 3 or above were barely seen among the three groups, with 14%, 36%, and 6% in the ADT plus abiraterone, ADT plus docetaxel, and ADT groups, respectively. Sixteen patients in the ADT plus docetaxel group experienced severe neutropenia, and the absolute neutrophil count (ANC) of the patients recovered to >1,000/mm^3^ after the injection of granulocyte colony-stimulating factor. Three patients had severely elevated ALT and/or AST in the ADT plus abiraterone group, and two discontinued the treatment. One patient’s liver enzyme level recovered to grade 1 within 1 month after hepatoprotective treatment, and neoadjuvant treatment was continued. There were no grade 5 AEs.

Operation-related data were comparable among the three groups, including operation time, blood transfusion rate, hospital stay, and perioperative complications.

## 4 Discussion

In our work, we evaluated the pathological response and post-RP outcomes for patients with very-high-risk prostate cancer (VHRPCa). Overall, ADT plus abiraterone or docetaxel could achieve better pathological response than ADT alone. ADT plus abiraterone was associated with better bPFS, whereas ADT plus docetaxel was not.

Despite ongoing efforts, there is no consensus regarding the optimal treatment for men with HRPCa. Numerous studies focusing on the treatment efficacy of neoadjuvant therapy on HRPCa showed no clear evidence of bPFS or OS advantages, indicating that the use of intense androgen deprivation therapy might be warranted (Schulman et al., 2000; Kimura and Egawa, 2018; McClintock et al., 2019; Liu et al., 2021). ADT combined with abiraterone has been proven to significantly lower intraprostatic androgen levels and reduce residual cancer burden (RCB) (Taplin et al., 2014; Efstathiou et al., 2019). The addition of abiraterone to enzalutamide and leuprolide also demonstrated higher rates of pCR or MRD, although the difference was not significant (McKay et al., 2019). However, it remains controversial whether the pathological endpoints can be surrogated for long-term benefits. Previous studies have discovered a 3-year biochemical recurrence-free survival (bRFS) ranging from 59.1% to 75.6% of combined therapy in the neoadjuvant setting whereas no direct comparisons were made between combined therapy and ADT alone (McKay et al., 2018; Karzai et al., 2021; Wilkinson et al., 2021). The wide range of bRFS might be interpreted by diverse treatment regimes, duration, and patient groups.

Even within HRPCa, oncological outcomes can be quite heterogeneous. Former studies mostly included unfavorable intermediate-risk and high-risk groups. One study carried out consisted of only 29% of participants with ≥ cT3 disease at initial diagnosis, and another study included only 24% (Taplin et al., 2014; McKay et al., 2019). Similar studies have also captured more favorable “high-risk disease” and scarcely focused on very-high-risk PCa (VHRPCa). We believe that the results for high-risk PCa in the neoadjuvant setting would be underestimated if the VHRPCa group was included. Our results present a superiority of the addition of abiraterone either in the pathological response or in biochemical progression-free survival (bPFS). It is worth noting that the majority of the cohorts (96.4%) in our study have VHRPCa. It is known that combining ADT with second-generation hormone treatment (abiraterone, enzalutamide, or apalutamide) improves the outcome of metastatic prostate cancer (Fizazi et al., 2017; James et al., 2017; Armstrong et al., 2019). It is reasonable to speculate that men with higher tumor burden may display more remarkable treatment effect with the addition of abiraterone. Further trials with a longer follow-up time and a larger sample size are certainly needed to confirm the speculation.

In addition, this is the first study to compare ADT plus abiraterone with ADT plus docetaxel as neoadjuvant therapy for HRPCa to the best of our knowledge. In our study, ADT plus docetaxel showed more strength in pathological response than ADT alone but not in bPFS. It suggested that ADT plus docetaxel might not be as efficient as ADT plus abiraterone in terms of survival, even though the difference is not significant (log-rank *p* = 0.177). Former research studies showed that docetaxel improves MFS and OS in metastatic patients but not in localized PCa (Fizazi et al., 2015; Rosenthal et al., 2019). Eastham et al. (2020) carried out a phase III randomized clinical trial comparing the effect of neoadjuvant chemo-hormonal therapy with RP alone. However, the primary endpoint of 3 year bPFS was not met. Notably, a higher incidence of AEs could be observed compared with the ADT plus abiraterone group. Considering adverse events, the preference for chemo-hormonal therapy would be further compromised.

Currently, non-surgical treatment remains the first-line strategy for VHRPCa (NCCN Clinical Practice Guidelines in Oncology NCCN Guidelines, 2022). The optimal management strategy is still unclear but is likely to involve a multimodal approach (Wilkins et al., 2020). Based on our results, radical prostatectomy (RP) and extended pelvic lymph node dissection (ePLND) following neoadjuvant therapy proved effective without additional severe AEs. Importantly, noticeable downgrading was observed, and complete surgical excision could be hopeful. In the ADT plus abiraterone group, 54% of participants had disease ≤ T2 at the final pathological specimen, while about 80% had extraprostatic extension at initial diagnoses. Exceptional pathological response (pCR or MRD) rates (31%) of intense neoadjuvant therapy were even more ideal than expected, comparable to that of intermediate- or high-risk PCa. The increased prevalence of HRPCa, advances in surgical techniques, and emergence of more potent anti-androgens have provided impetus for researchers to consider surgery as part of multimodal therapy. With favorable pathological response, well-designed phase III trials including *PROTEUS* and SHR3680 were promising to provide future perspectives and improve prognoses for HRPCa and VHRPCa.

There are also some limitations to this study. Even with comparable baseline characteristics among the three groups, the results of this pooled analysis might be compromised due to the lack of direct randomization. Another limitation is the relative short follow-up time. The median follow-up time is 42 months, and median bPFS for ADT plus abiraterone has not yet been reached. Thus, research studies with longer follow-up time and OS or MFS endpoints are needed to further confirm the long-time survival benefits. In addition, subgroup analysis regarding tumor immunocytochemistry characteristics might be better conducted to explore the potential mechanism of differing pathological response or clinical outcomes.

## 5 Conclusion

As neoadjuvant therapy for VHRPCa, ADT plus abiraterone or docetaxel could achieve better pCR or MRD rates than ADT alone. Longer bPFS was observed in ADT plus abiraterone but not in ADT plus docetaxel. The ADT plus abiraterone or docetaxel appeared tolerable.

## Data Availability

The original contributions presented in the study are included in the article/Supplementary Material; further inquiries can be directed to the corresponding authors.
