# Supplementary material for: Androgen deprivation therapy plus abiraterone or docetaxel as neoadjuvant therapy for very-high-risk prostate cancer: a pooled analysis of two phase II trials
Source: Front Pharmacol. 2023 Jun 26;14:1217303. doi: 10.3389/fphar.2023.1217303 (PMC10331422; doi:10.3389/fphar.2023.1217303)
Supplement: Supplementary file 1 [file Table2.DOCX]

**SUPPLEMENTARY MATERIALS**

**Table S1.** **Multivariable Cox regression model for bPFS**

| **Factor** | **Univariate** | | **Multivariate** | |
| --- | --- | --- | --- | --- |
|  | HR(95% CI) | *p* | HR(95% CI) | *p* |
| Group |  |  | - | - |
| Docetaxel vs ADT | 0.70(0.40-1.22) | 0.205 | 0.64(0.37-1.11) | 0.110 |
| Abiraterone vs ADT | 0.46(0.25-0.85) | 0.013^*^ | 0.44(0.23-0.83) | 0.011^*^ |
| Age | 0.99(0.95-1.03) | 0.670 | - | - |
| Initial PSA | 1.00(1.00-1.00) | 0.146 | - | - |
| Biopsy Gleason | 1.18(0.97-1.43) | 0.093 | - | - |
| Initial T stage | 1.25(0.98-1.60) | 0.078 | - | - |
| Initial volume | 0.99(0.97-1.01) | 0.354 | - | - |
| Risk stratification | 1.63(0.87-3.41) | 0.197 | - | - |
| Post-treatment volume | 0.99(0.96-1.02) | 0.623 | - | - |
| Post-treatment PSA | 1.63(0.78-3.41) | 0.197 | - | - |
| pCR or MRD | 0.42(0.21-0.85) | 0.016^*^ | - | - |
| Pathological margin | 5.57(3.29-9.44) | <0.001^**^ | 4.75(2.71-8.33) | <0.001^**^ |
| Pathological N stage | 2.06(1.14-3.72) | 0.016^*^ | - | - |
| Pathological T stage | 1.73(1.35-2.22) | <0.001^**^ | 1.49(1.12-1.97) | 0.006^**^ |

a. Group, age, initial PSA, Gleason, initial T stage, initial volume, risk stratification, post-treatment PSA, post-treatment volume, pCR or MRD, pathological margin, pathological N stage, and pathological T stage were included in the Cox regression.

b. For T stage, T2, T3a, T3b, and T4 were assigned 1, 2, 3, and 4, respectively, and were included in the analysis as continuous variables.

^*^p<0.05. ^**^p<0.01.

bPFS: biochemical progression-free survival; HR: hazard ratio; CI: confidence interval; ADT: androgen deprivation therapy; pCR: pathological complete response; MRD: minimal residual disease.

**Table S2. Adverse events** **with incidence ≥10% in any group**

| **Adverse events with incidence ≥10% in any group, n (%)** | **ADT (N=50)** | | **ADT plus**  **Docetaxel (N=50)** | | **ADT plus**  **Abiraterone (N=50)** | |
| --- | --- | --- | --- | --- | --- | --- |
|  | Any grade | Grade 3-4 | Any grade | Grade 3-4 | Any grade | Grade 3-4 |
| Hot flash | 23 (46) | 2 (4) | 22 (44) | 1 (2) | 25 (50) | 1 (2) |
| Granulocytopenia | 0 | 0 | 37 (74) | 16 (34) | 5 (10) | 0 |
| Fatigue | 8 (16) | 1 (2) | 19 (38) | 1 (2) | 7 (14) | 0 |
| Anemia | 5 (10) | 0 | 25 (50) | 2 (4) | 3 (6) | 0 |
| Hypokalemia | 0 | 0 | 0 | 0 | 28 (56) | 1 (2) |
| Gastrointestinal discomfort | 8 (16) | 0 | 9 (18) | 1 (2) | 8 (16) | 2 (4) |
| Hyperglycemia | 2 (4) | 0 | 3 (6) | 0 | 18 (38) | 0 |
| Thrombocytopenia | 0 | 0 | 18 (36) | 2 (4) | 1 (2) | 0 |
| Elevated AST | 2 (4) | 0 | 3 (6) | 1 (2) | 12 (24) | 3 (6) |
| Elevated ALT | 3 (6) | 0 | 3 (6) | 1 (2) | 9 (18) | 2 (4) |
| Hypertension | 3 (6) | 0 | 3 (6) | 0 | 8 (16) | 0 |
| Arthralgia or myalgia | 3 (6) | 0 | 5 (10) | 0 | 6 (12) | 0 |
| Fluid retention/edema | 5 (10) | 0 | 4 (8) | 0 | 3 (6) | 0 |
| Infection | 2 (4) | 0 | 6 (12) | 1 (2) | 3 (6) | 1 (2) |
| Neuropathy | 0 | 0 | 5 (10) | 1 (2) | 0 | 0 |
| Allergic reaction | 1 (2) | 0 | 2 (4) | 1 (2) | 1 (2) | 0 |

a. Adverse events reported according to National Cancer Institute Common Terminology Criteria for Adverse Events version 5.0.

b. AEs were shown when in ≥10% of patients in either treatment group or when any grade ≥3 events occurred. No grade 5 events were observed.

c. Two patients in the abiraterone group discontinued treatment due to severe elevations in ALT and/or AST.

ADT: androgen deprivation therapy; AST: aspartate aminotransferase; ALT: alanine aminotransferase.

**Table S3.** Post-biochemical recurrence treatment

|  | **ADT**  **(N=25)** | **ADT plus Docetaxel**  **(N=26)** | **ADT plus Abiraterone**  **(N=17)** |
| --- | --- | --- | --- |
| ADT | 11 | 14 | 9 |
| Radiation | 10 | 9 | 6 |
| Radiation +ADT | 4 | 3 | 2 |
| Combined with antiandrogens | 8 | 6 | 4 |

ADT = androgen deprivation therapy.

**Table S4.** Pre-treatment and Post-treatment ^68^Ga-PSMA-11 PET/CT related information

|  | **ADT**  **(n=20)** | **ADT plus**  **Docetaxel (n=31)** | **ADT plus**  **Abiraterone (n=31)** | **Total (n=82)** ^@^ |
| --- | --- | --- | --- | --- |
| **Pre-treatment** |  |  |  |  |
| Tumor volume, ml, Median (IQR) ^%^ | 9.64 (3.00-18.20) | 8.22 (4.50-19.40) | 14.31 (6.30-21.15) | 11.35 (5.95-20.00) |
| SUVmean, Median (IQR) | 7.75 (5.23-10.78) | 7.30 (5.70-10.60) | 8.55 (6.70-12.03) | 7.90 (5.75-11.10) |
| SUVmax, Median (IQR) | 24.15(12.05-45.18) | 19.40(12.30-34.30) | 23.95(16.70-46.83) | 22.90 (13.15-44.30) |
|  |  |  |  |  |
| **Post-treatment** |  |  |  |  |
| Tumor volume, ml, Median (IQR) | 5.50 (2.00-6.85) | 3.10 (1.80-5.60) | 2.53 (0.82-6.17) | 3.00 (1.20-6.15) ^#^ |
| SUVmean, Median (IQR) | 5.35 (3.80-6.48) | 3.75 (2.90-4.60) | 4.00 (3.05-4.93) | 4.10 (3.20-5.20) ^&^ |
| SUVmax, Median (IQR) | 12.3 (5.68-19.18) | 6.90 (5.00-9.20) | 6.20 (4.18-7.80) | 6.60 (4.90-11.58) ^$^ |

@ 20, 31 and 31 patients received ^68^Ga-PSMA-11 PET/CT examination both before and after treatment. There was no significant difference among groups (*p*=0.150).

% Tumor volume was determined by multiplying the sum of the areas by section thickness (3mm) and by a 1.5 correction factor for tissue shrinkage during processing.

# ^68^Ga-PSMA-11 PET/CT estimated post-treatment tumor volume decreased significantly compared with pre-treatment (*p*<0.01).

& Post-treatment SUVmean decreased significantly compared with pre-treatment (*p*<0.01).

$ Post-treatment SUVmax decreased significantly compared with pre-treatment (*p*<0.01).

Partial participants underwent both pre-treatment and post-treatment ^68^Ga-PSMA-11 PET/CT imaging, which was performed using an uMI 780 PET-CT scanner (United Imaging Healthcare (UIH), Shanghai, China). All images were normalized to decay corrected injected activity per kg body weight (SUV [g/ml]). SUVmean and SUVmax of a tumor lesion was achieved by a PET/CT post-processing software.

ADT = androgen deprivation therapy; SUV = standard uptake value.

**Table S5.** ^68^Ga-PSMA-11 PET/CT features correlated with pCR

|  | **pCR (n=12)** | **Non-pCR (n=70)** | ***p*** |
| --- | --- | --- | --- |
| **Pre-treatment** |  |  |  |
| Tumor volume, ml, Median (IQR) | 13.45 (4.30-21.00) | 10.90 (5.95-20.84) | 0.052 |
| SUVmean, Median (IQR) | 7.85 (5.73-10.14) | 8.00 (5.78-11.55) | 0.529 |
| SUVmax, Median (IQR) | 20.55 (13.75-39.60) | 23.95 (13.18-35.08) | 0.312 |
|  |  |  |  |
| **Post-treatment** |  |  |  |
| Tumor volume, ml, Median (IQR) | 0.25 (0.00-1.55) | 3.85 (1.95-6.50) | <0.001^**^ |
| SUVmean, Median (IQR) | 3.25 (2.50-3.58) | 4.40 (3.35-5.55) | 0.001^**^ |
| SUVmax, Median (IQR) | 4.65 (4.13-5.80) | 7.60 (5.35-11.90) | 0.001^**^ |

^**^ *p*<0.01.

ADT = androgen deprivation therapy; SUV = standard uptake value.


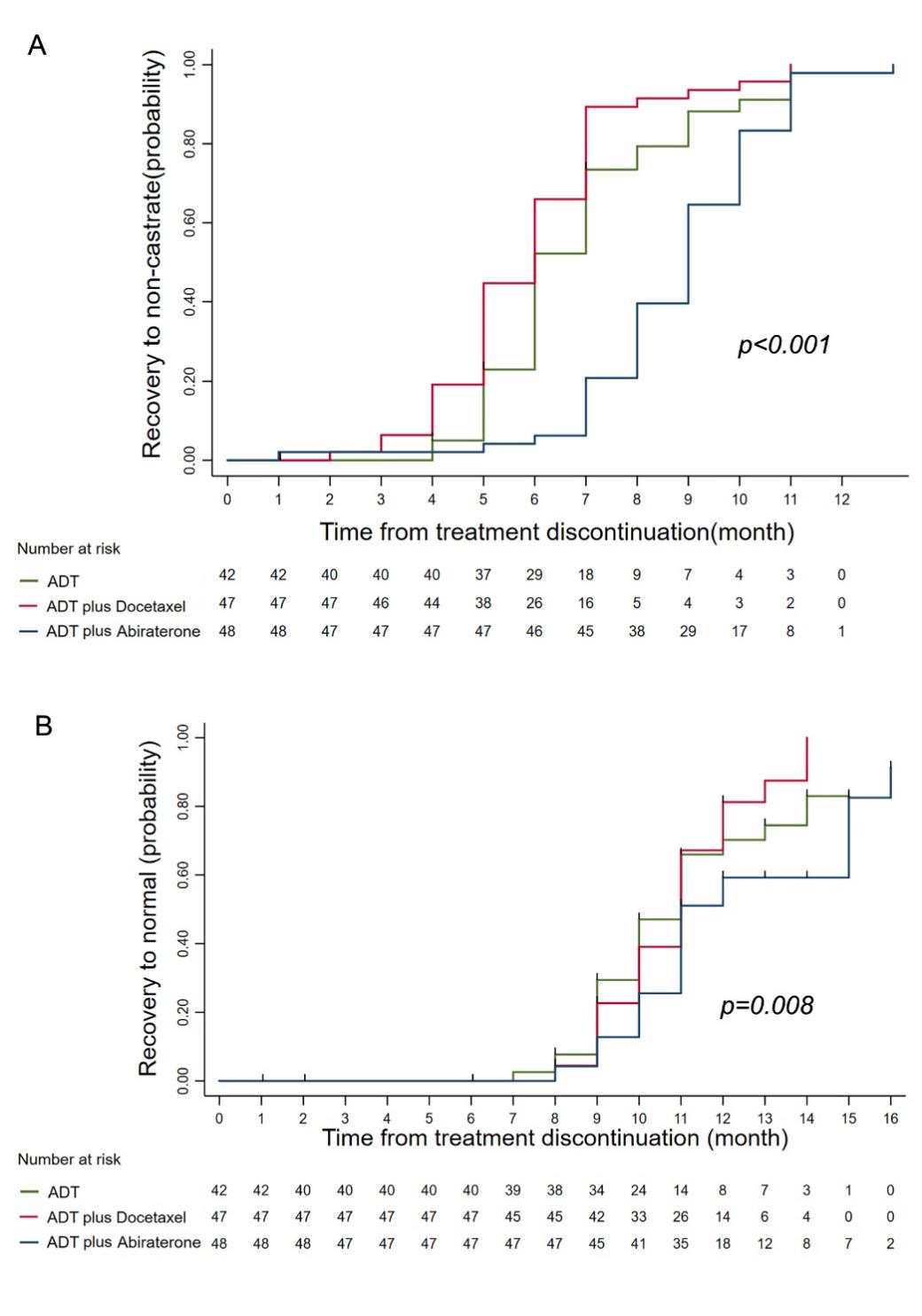


**Figure S1**. Testosterone level recovery from treatment discontinuation

(A) The figure shows the testosterone recovery to non-castrate level (≥ 50ng/dl) from treatment discontinuation. The overall median recovery time is 7 months and the median recovery time for ADT, ADT plus Docetaxel and ADT plus Abiraterone is 6, 6, 9 months respectively. Significant difference is found between groups (*p*<0.001). (B) The figure shows the testosterone recovery to normal level (≥ 300ng/dl) from treatment discontinuation. The overall median recovery time is 11 months. Though the ADT, ADT plus Docetaxel and ADT plus Abiraterone demonstrate the same median recovery time, significant difference is still found between groups (*p*=0.008).


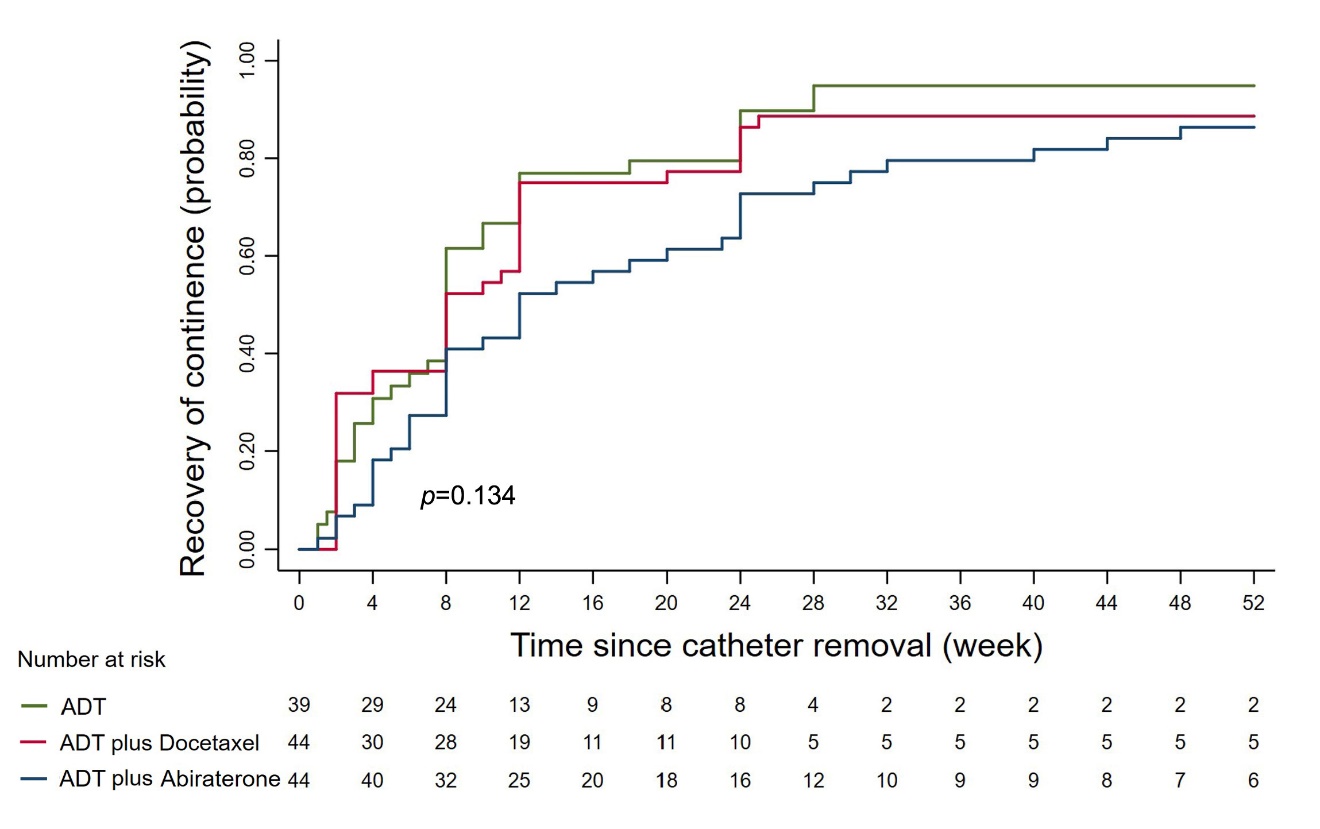


**Figure S2**. Post-RARP urinary continence recovery

The figure shows the recovery of urinary continence since removal of catheter after Robot-assisted radical prostatectomy (RARP). The overall median time to restore continence is 8 weeks. No difference is found between ADT, ADT plus Docetaxel and ADT plus Abiraterone group (*p*=0.134).

Operation related data were comparable among the three groups, including operation time, blood transfusion rate, hospital stay and perioperative complications (p<0.05). The average operation time of the patients was 211±39 min. The average intraoperative blood loss was 128±35 ml. The blood transfusion rate was 7.3%. The average postoperative hospital stay was 4.0±1.2 days. Postoperative complications mainly included 12 cases of lymphatic leakage (four, three, and five in the ADT, ADT plus docetaxel, and ADT plus abiraterone groups), one case of anastomotic leakage (ADT plus docetaxel group), 18 cases of obturator nerve injury (three, eight, and seven in the ADT, ADT plus docetaxel, and ADT plus abiraterone groups; all of which were transient nerve injuries and recovered within 1 month after the operation), and one case of rectal injury which further underwent rectal repair without colostomy. No iliac vessel injury occurred.
